# Ovation of biopolymers in conterminous EU members *via* clustering of biotechnological advances : A mini-compendium

**DOI:** 10.3389/fbioe.2022.1061652

**Published:** 2022-12-01

**Authors:** Spyridon Achinas, Efthymios Poulios, Simon Bergsma, Gerrit Jan Willem Euverink

**Affiliations:** ^1^ Faculty of Science and Engineering, University of Groningen, Groningen, Netherlands; ^2^ 4th Department of Surgery, Attikon University Hospital, Medical School, National and Kapodistrian University of Athens, Athens, Greece

**Keywords:** biopolymers, bioplastics, synthetic biology, bioeconomy, biomaterials

## Introduction

The yawning gap between green bioproducts and fossil-based products calls into question whether the chemical industry can underpin the drive for sustainable development and reduction of socio-ecological disparities. The biochemical industry has, ostensibly, struggled with technological barriers. A wide assortment of biomedical and biopharmaceutical applications, ranging from implant fabrication to drug delivery, can be attributed to biopolymers (Baranwal et al., 2022; Pathak et al., 2022).

The development of biopolymers is mired in contradictions regarding the polymeric molecules. Biopolymers are polymeric biomolecules produced by living organisms, such as plants or bacteria. These types of polymers can be produced from renewable sources, are biodegradable, and can, therefore, be recycled by biological processes. However, certain chemical-based synthetic polymers are toxic to human and animal cells. Thus, biopolymers have been considered pinch hitters in biomedical and biopharmaceutical applications (Baranwal et al., 2022; Pathak et al., 2022).

At present, the biochemical projects have stumbled due to operational barriers and remain uncertain in light of market conditions. Getting these projects on the front burner is crucial for the establishment of a healthy market environment, where applicational infrastructure is considered a catalyst in fostering the evolution of biopolymers. Breakthroughs in synthesis and the advanced properties of biopolymers are intrinsic pillars for the expansion of biomedical applications, including their use in anti-inflammatory and antimicrobial agents, drug delivery systems, and regenerative medicine (tissue engineering and implant fabrication) (Scalzone et al., 2020; Peng et al., 2022).

In this article, we explore subtle issues of the bio-based product landscape and suggest steppingstones towards biopolymer production. This mini compendium provides a timely discussion of the ongoing debate regarding biopolymers.

## Cradle-to-cradle biopolymer strategy

The development of sustainable solutions is essential to drive the radical change to a green, bio-based economy. The socio-ecological deadlock reflects a cross-cutting concern about drastic change. The European Commission rendered a sharp rebuke to European Union (EU) members (European Biopolymers Market, 2022b), imploring them to embrace biopolymer projects by accruing supplementary budgeting for investment in bio-based products (Mense and van Kootwijk, 2021). Against that backdrop, there is room for manoeuver and companies are enamoured with sustainable bio-based solutions. Biochemical business leaders are trying to be savvy in addressing the major threat to their activities; societal concerns and call for an abrupt curtailment of chemical consumption is an existential risk for the companies. The establishment of European consortia is regarded as a crucial impetus for the companies to consider climate change, invest substantial funding in green polymers, and thereby expedite the transition to a sustainable bioeconomy. The European Commission has developed reforms that place obligations on EU members to ensure environmentally sound treatment of biowaste.

Currently, the legislation includes the waste framework directive 2018/851, as indicated by EU commissions directives, which provides a long-term roadmap for waste elimination and bio-product recovery (EUR-Lex, 2018). Plastics are covered in annex 1 of the European strategy for plastics in a circular economy, and communications linked to the EU waste policy and circular economy include the European Green Deal, 11-12-2020, and the Circular Economy Action Plan 2.0, 11-3-2020 (EUR-Lex-b, 2022). The purposes of these communications are to improve regulations for biowaste collection and management, enhance advanced technology infrastructure, and drive a dedicated biorecycling and waste-derived polymer market (Mense and van Kootwijk, 2021). EU policy is critical to encourage companies to reduce their waste and support recycling initiatives. Stakeholders in the biochemical industry, including government authorities, industrial experts, and local communities, have stepped up their efforts to develop a firm strategy/policy framework that reduces inconsistencies in the waste management arena and addresses the key priorities of the waste-to-biopolymer sector. Biochemical companies have ramped up production of waste-derived products, including biopolymers. For many, the appeal of value-added intermediate products may reinforce the efficient use of waste (Ashokkumar et al., 2022).

**FIGURE 1:**
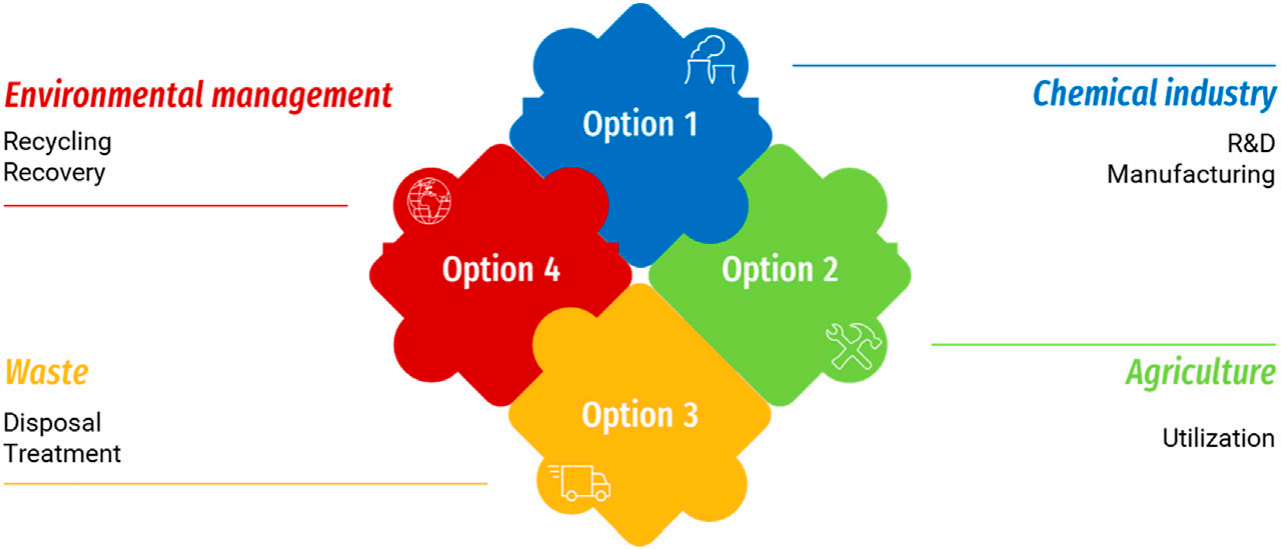
Lifecycle of biopolymers in the agricultural industry.

An example of the lifecycle of bio-based, biodegradable, and compostable plastics, with the aim of redeeming quality and value, in the economic reality of agriculture is given in Figure 1. The environmental management concept is an evolving scheme that includes technological advancements, product applicability, dedication to sustainability, and governmental involvement. Biopolymers are a promising way to attenuate the effects of fossil-based, non-biodegradable plastics in the environment.

Polymer companies pushing for more transparency in the bioproduct system question the claim that this is a sustainable alternative to chemical-based polymers. The R&D department focuses on churning out new biopolymer synthesis methods tailored to achieve a sustainable and efficient cascading technological route (Ladiè et al., 2021; Masek and Kosmalska, 2022). Although transition to a bio-based economy has barriers and challenges, unequivocal and plausible policy is crucial for economic transformation and support of the creativity necessary to overcome the critical factors governing large-scale operations (Mense and van Kootwijk, 2021).

## Synthetic genomics echoing biopolymers revolution

The advent of genomics resuscitated the biopolymer industry and rekindled the ailing bioeconomy. Microorganism evolution hinges on the complex interplay between microbiomes and environmental conditions. A cardinal number of biopolymer-related studies and goals delineate the status quo; it has been extensively alluded that utilisation of microbiological agents genetically modified to produce bioresources will benefit the biopolymer industry (Carruthers and Lee, 2022).

Current research efforts in synthetic biology, as well as a bewildering number of reports on the weaning from chemical-based polymers, may herald unprecedented industrial focus. Pioneering work in genomics has also made possible the industrial stance regarding advanced bioproducts. Synthetic biology demonstrates the shrewdness of the biotech industry in embedding laboratory techniques and leveraging data and technologies (Zheng et al., 2020; Asiri et al., 2022).

The ecological deadlock reflects a cross-cutting alarm and need for a proactive stance with drastic alterations in this direction. Synthetic biology is necessary to perk up the landscape and provide a cushion in times of food chain disruption. Future ventures that provide more funding of research may reinforce the bio-based product innovation (Nanda et al., 2022). The reputation of chemical technologies is declining in the face of social and environmental awareness that attenuates their competitiveness.

Environmental organizations suggest that bioeconomy-based research may curtail the production of inordinate amounts of emissions. The race to a bio-based economy requires collaboration between venture capital firms and scientific institutions. Investors and corporations are enamoured with technological breakthroughs and scientific advances. However, curtailments in the course of large-scale applications for bio-based production slow their commercialization (Rodriguez-Perez, et al., 2018). Efforts are continued to ensure the priority of environmental effects in establishment and maintenance of a green economy and sustainability practises; all of this while industrialization challenges the implementation of biopolymer technologies and the expansion of the portfolio of available bio-based technologies (Sanchez-Salvador et al., 2021). Nevertheless, efforts to commercialize are futile due to the reluctance to circumvent political roadblocks and the delays having negative effects on the biopolymer industry.

## Realising the value of biowaste in the biopolymer industry

Leaders in the biochemical industry are considering waste as a sustainable solution for biopolymer production. Biowaste is regarded as a new biosource, related to biopolymers, which may be respected in the business arena. The promise of genetically engineered crops and algae is tantalizing, as previous studies have shown that genetic manipulation of organisms results in efficient secretion of biopolymeric compounds (Moretto et al., 2020). Bolstered efforts for sustainable biochemical recovery are conceivable; however, eco-friendly and profitable practises to abate biowastes are needed to reroute the economy on a sustainable trajectory (Teixeira-Costa and Andrade, 2021; Iqbal et al., 2022). Several chemical companies are experimenting with, and advocating for, new waste and recycling infrastructure strategies.

In addition, the annual amount of generated waste is dwarfed by the impact of national, regional, and local waste. Idleness on the part of several European members highlights the necessity for market reformation and stringent regulation; national policies need to converge on sustainable biochemical recovery in order to deal with waste accumulation. The resounding agreement on substitution of biowaste for waste provides grounds for complacency among biopolymer business investors (Ashokkumar et al., 2022).

As expected, environmental pollution is greatest in cities, as is the vigilance in reduction of biowaste, thereby creating a sweeping basis for reliable biochemical investments with the guarantee of long-term contracts (Ranganathan et al., 2020). Prioritization of biopolymers by the EU has upped the *ante* by imposing elevated market standards to spur the biochemical industry.

## Questioning the technological findings

The clustering of biowaste to biochemical technologies is a prerequisite for the sustainable production of biopolymers. Manoeuvrability of the technical pathways circumvents conventional chemical recovery and poor end-product availability (Nanda et al., 2022). Policy is a crucial component to expedite the application of advanced technologies through which to begin treading the circular bioeconomy path (Ashokkumar et al., 2022).

However, the peculiarities of current policy result in energy businesses going bankrupt and the waste-to-energy sector becoming caught in a minor/major economic depression. Leveraging new technologies will spark change towards an eco-friendly industrial scheme with business risk averseness expediting sustainable development. Research advancements can offer a new lease on life to the biochemical sector. The European Commission must reveal the proximity of the biochemical market and end the unequal distribution of European subsidies to sustain stricken biopolymer producers and beleaguered biochemical companies.

One of the main goals of the EU is to bail out low GDP EU members that suffer from significant economic austerity with regard to sustainable infrastructure. Technological breakthroughs in waste treatment techniques, referred to by some as the critical juncture of the biochemical economy, have led to the pledge that bioprocess scientists will expand the scope of end-products generated from waste treatment activities. Contentious arguments over the clinical translation of biomaterials (animal protein-based extracted biopolymers) concomitantly jeopardise the commercialization of these techniques. As suggested by the results of several studies, understanding the operational factors may improve high-temperature technologies.

When new bioprocessing technologies become mainstream, there is inevitable deliberation about their sustainability and efficiency prior to market integration. These dilemmas galvanise the debate surrounding fossil-based and green polymers. As a result, chemical companies are edging away from their main fossil-based products and are contending with a surge of investments in biopolymer projects.

However, there is scepticism within the chemical industry about whether it is possible to anchor their activities in green plastics (Aurisano et al., 2021). The consensus is that ‘‘billions of euros would be needed’’ to phase out fossil-based plastics and fully replace the existing synthetic methods with alternative-bioprocessing techniques (Zheng and Suh, 2019). The plastic industry contemplates the biowaste issue whilst being shunned by environmental associations. Major biopolymers construction projects are underway; however, uncertainty amongst those in the chemical industry and heedless investments can imperil project viability. Plausible technological experience is needed to dodge operational risks and implement sustainable business practises with synergy and alacrity, even amidst members on the sidelines, to prevent the bioeconomy from imploding.

The biopolymer industry’s attempts to keep pace have been ineffective due to limited financial support from the government, dubious applicability, and investor apathy (Baranwal et al., 2022). Researchers have focused on identification and testing of efficient synthetic methods to produce biopolymers. Therefore, niche technology is often held up at the lower levels of development due to a lack of supportive policy. A reliable, well-implemented policy would bolster the infrastructure of the biochemical industry and prevent further investments in fossil-based polymers over the next decade (Mense and van Kootwijk, 2021). Continuing government subsidies buoy the biochemical industry, allowing it to manage the ebbs and flows of bioplastic production capacity. An erratic legal framework is holding the industry back from potential aid, thereby preventing the elimination of carbon emissions and amortisation for past climate change pitfalls. Despite the deluge of press and research releases insisting that bioplastics are imminent, the fact remains that no institutional investor has yet figured out how to ramp up allocations to biochemical infrastructure. Instead, biochemical producers have received significant funding to pursue the elusive goal of producing biopolymers from biowaste.

## Economy

Biopolymers encounter sizable barriers to entry and ascension in the polymer economy. Their cost of production eclipses the costs of the production of conventional polymers by 2.5–7.5 times (Coherent Market Insights, 2022). In addition, infrastructure for mass production is still lacking compared to that of conventional petroleum-based polymers. Due to the nascent stage of biopolymer production, innovation is critical. Innovation will be the key to driving down production costs and enhancing the competitiveness of biopolymers. These innovations require significant resources and therefore investments from both companies and governments. Increasingly stringent regulations on conventional plastics, as laid out by the European Commission, could also propel the biopolymer market.

In 2021, the annual bioplastic production in Europe was 0.58 million tonnes (European Bioplastics). The worldwide COVID-19 pandemic had profound effects on industries and consumer behaviour. Logistical issues and material and manpower shortages have plagued industry and contracted or wiped-out economic growth. The plastic market was also strongly affected. Packaging is the largest and fastest growing market for biopolymers. Packaging deemed unnecessary was hit hard by the pandemic. However, the pandemic induced a rise in e-commerce and initiated new demand for packaging in essential shipping. A rapid paradigm-shift to the digital age was induced by this global crisis. Companies, to remain competitive, will have to evolve rapidly against this backdrop.

Data show that the European market for biopolymers is rapidly expanding, and forecasts indicate the market value is set to increase from 1.2 billion dollars in 2021 to 3.4 billion dollars in 2026 (European Biopolymers Market, 2022a). Overall, the biopolymer market is showing strong growth. EU directives, industry interest amplified by consumer awareness, and an increase in scientific innovation powered by investments have all contributed to the upsurge of biopolymers.

## Conclusion

This mini compendium described and discussed the biopolymer conundrum and the repercussions for the bio-based economy. The European Union aims to implement a cascade strategy towards the synthesis of biopolymers to address the challenges posed by plastics throughout the value chain. Technological findings and innovations may improve the value chain functionality and drive investments in the right direction. The pouring of millions of Euros and dollars into biopolymers has enticed companies in industries from agribusiness and biotechnology to reduce use of petroleum and chemicals. The allure of a sustainable and greener future has solidified the preferences of investors and bystanders who pin their hopes for the world’s energy future on biopolymers.
